# COVID-19 vaccination may cause FDG uptake beyond axillary area

**DOI:** 10.1186/s41824-021-00105-2

**Published:** 2021-06-01

**Authors:** Vincent Fleury, Bruno Maucherat, Daniela Rusu, Frédéric Dumont, Caroline Rousseau

**Affiliations:** 1 Department of Nuclear Medicine, ICO Gauducheau Cancer Center, Boulevard Monod, 44805 Saint-Herblain Cedex,, France; 2 Department of Surgical Oncology, ICO Gauducheau Cancer Center, Saint-Herblain, France; 3 CNRS, Inserm, CRCINA, Nantes, France; 4 Nantes University, Nantes, France

**Keywords:** FDG PET/CT, COVID-19, Vaccine

## Abstract

**Background:**

The vaccination immune response may induce false-positive ^18^F-FDG PET/CT uptake.

**Case presentation:**

An extended supraclavicular lymph nodal activation after coronavirus disease 2019 (COVID-19) vaccination revealed on ^18^F-FDG PET/CT mimics a Virchow nodule in a patient with medical history of well-differentiated appendicular adenocarcinoma.

**Conclusion:**

This case highlights a nodal activation beyond axillary area and the importance of documenting vaccination history at the time of scanning to avoid false-positive results.

## Background

Development of vaccines to prevent COVID-19 is a hope to prevent transmission or reduce the severity of infection. However, vaccination could be a potential source of false-positive results in ^18^F-FDG PET/CT (Katal et al. 2021).

## Case presentation

**Fig. 1:**
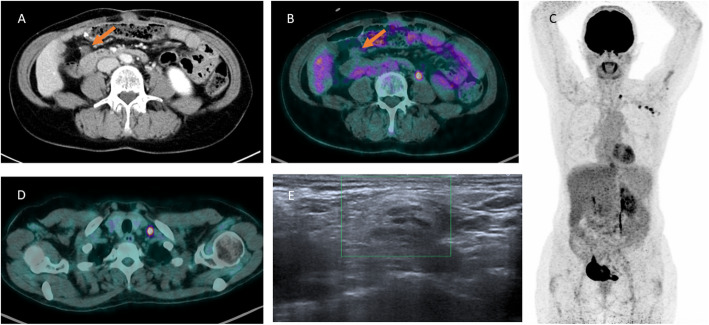
CT scan showed a peritoneal nodule (arrow in **a**) with no increased uptake on fused ^18^F-FDG PET/CT images (arrow in **b**). MIP (**c**) and fused ^18^F-FDG PET/CT (**d**) showed with increased uptake on the left axillary lymph nodes up to the left supraclavicular area. Echography (**e**) showed a normal supraclavicular lymph node

We present the case of a 64-year-old female with well-differentiated appendicular adenocarcinoma associated with peritoneal carcinosis initially treated by surgery and chemotherapy benefited from a ^18^F-FDG PET/CT to investigate a peritoneal nodule (Fig. 1). This peritoneal nodule (arrow) visualized on the axial (**a**) view of CT image showed no increased FDG uptake on the axial (**b**) PET/CT fused image. The MIP (**c**) and axial (D) PET/CT fused images detected an intense hypermetabolism on the left axillary lymph nodes up to the left supraclavicular area. The patient revealed she had received the first of dose Pfizer BNT162b2mRNA vaccine against COVID-19 on the left shoulder intramuscular 4 days before FDG examination. In order to exclude a Virchow nodule due to her digestive cancer history, we performed a cervical echography with supraclavicular node cytological biopsy sample. Echography (E) showed a 14-mm-long axis normal lymph node with its central hilum. Cytological analysis revealed activated lymphoid cells without tumor cells.

## Discussion

Several previous reports have demonstrated axillary lymph nodal activation on ^18^F-FDG PET/CT following influenza and COVID-19 vaccination (Burger et al. 2011; Shirone et al. 2012; Eifer et al. 2021; Nawwar et al. 2021). This case revealed an atypical extended supraclavicular activation. In the context of the COVID-19 pandemic and large vaccination programs, questionnaires including date and location of the vaccination can help to avoid false-positive lymph node interpretation with the risk of a therapeutic choice impact offered to the patient. In patients with solid tumor like breast cancer or melanoma, the vaccination should be performed in the contralateral arm to limit misinterpretations. Otherwise, it would be advisable to respect a time interval to define between the vaccination and ^18^F-FDG PET/CT scan.

## Conclusion

Nuclear physicians should be careful when cancers staging and re-staging. This is especially important for patients with breast cancer having been vaccinated on the homolateral upper limb, digestive cancer patients vaccinated on the left side, or with lung or head and neck carcinoma.

## Data Availability

Not applicable
